# Supplementary material for: Evaluation of usefulness in surfactant protein D as a predictor of mortality in myositis-associated interstitial lung disease
Source: PLoS One. 2020 Jun 11;15(6):e0234523. doi: 10.1371/journal.pone.0234523 (PMC7289364; doi:10.1371/journal.pone.0234523)
Supplement: Attachment — Submitted filename: Responce to Reviewers comments.docx [file pone.0234523.s001.docx]

Dear Editor and Reviewers

Thank you for giving us your thoughtful comments/suggestions. We have responded to them one by one, and revised our manuscript according to the manner as below. We are looking forward to your positive consideration of the publication in our manuscript.

Best regards,

Takahisa Gono, corresponding author

**Reviewer #1:**
*1. I agree with authors lower SP-D levels is potentially relevant to the rapid progressive course of anti-MDA5 antibody-associated ILD and the level could be low at the disease diagnosis. However, is there any speculation on the issue which patients with anti-ARS had a higher level of SP-D among other MSAs? Since the elevated serum levels of SP-D could be attributed to an increase in SP-D production by regenerating alveolar type II pneumocytes, did the patients with anti-ARS have already widespread findings of ILD on chest CT at diagnosis or have longer disease duration from diagnosis of ILD?*

**Response:** I appreciate your valuable comments. As the reviewer suggested, the elevated serum levels of SP-D could be dependent on the degree of regenerating alveolar type II pneumocytes and/or that of destruction in alveolar capillaries. To answer the reviewer’s suggestions, we compared disease duration at diagnosis of ILD among three groups: anti-MDA5 antibody-positive group, anti-ARS antibody-positive group and, double-negative group. The median disease duration (interquartile range [IQR]) was 2 months (1-3), 3 months (1-8), and 3 months (1-6) in anti-MDA5 antibody-positive group, anti-ARS antibody-positive group and, double-negative group, respectively. The disease duration was significantly shorter in anti-MDA5 antibody-positive group than in anti-ARS antibody-positive group (*P* = 0.0002) or double-negative group (*P* = 0.0005). The range of disease duration in anti-ARS antibody-positive group was 0-122 months; longer than that of double-negative group with 0-88 months, although there was no significant difference (*P* = 0.82) between the two groups.

In terms of the severity of pulmonary dysfunction with %vital capacity (VC), the median value (IQR) of %VC was lower in anti-ARS antibody-positive group: 70.2% (60-85), than in double-negative group: 84.9% (66-98) (*P* = 0.0002) or anti-MDA5 antibody positive group: 81% (66-98) (*P* = 0.0005) (revised Figure 2B). On the other hand, there was no significant difference (*P* = 0.53) between double-negative group and anti-MDA5 antibody-positive group. Unfortunately, we have no data regarding chest HRCT score.

These findings indicated that the serum levels of SP-D in anti-ARS antibody-positive group were influenced by more widespread damage of ILD as well as the longer disease duration of ILD.

To describe this point, we have added the following sentences as below.

In line 168 to 182 on page 7 to 8,

“We also compared disease duration and pulmonary function among the three groups. With regard to disease duration at diagnosis of PM/DM-associated ILD, the median disease duration (IQR) was significantly shorter in anti-MDA5 antibody-positive group: 2 months (1-3), than in anti-ARS antibody-positive group: 3 months (1-8) (*P* = 0.0002) or double-negative group: 3 months (1-6) (*P* = 0.0005). The range of disease duration in anti-ARS antibody-positive group was 0-122 months; longer than that of double-negative group with 0-88 months, although there was no significant difference of the median disease duration (*P* = 0.82) between the two groups. In terms of the severity of pulmonary dysfunction with %vital capacity (VC), the median value (IQR) of %VC was lower in anti-ARS antibody positive group: 70.2% (60-85), than in double-negative group: 84.9% (66-98) (*P* = 0.0002) or anti-MDA5 antibody positive group: 81% (66-98) (*P* = 0.0005) (Figure 2B). On the other hand, there was no significant difference (*P* = 0.53) between anti-MDA5 antibody-positive group and double-negative group. These results indicated more widespread damage of ILD as well as longer disease duration in anti-ARS antibody-positive group than the other two groups.”

In line 222 to 229 on page 9,

“Histopathological differences of ILD at diagnosis of PM/DM-associated ILD might contribute to the behavior of serum SP-D levels in each MSA group. In this study, the disease duration was significantly longer in in anti-ARS antibody-positive group or double-negative group than in anti-MDA5-positive group. In addition, the median value of %vital capacity was lower in anti-ARS antibody-positive group than in double-negative group or anti-MDA5 antibody-positive group. These findings might indicate that chronic damage and remodelling of peripheral pulmonary structure was more apparent in anti-ARS antibody-positive group, causing translocation of SP-D molecules from alveolar epithelium to blood vessels much more.”

**Reviewer #2:**

*1. Please provide intra-class comparison of non-survivor vs. survivor in each MSA group.*

**Response:** Please see Figure 1B, 1C and 1D. We have provided the intra-class comparison of serum SP-D levels between non-survivors and survivors in each MSA group.

*#2 It should be emphasized that serum SP-D is approved as a laboratory test for clinical use and easily available at clinical practice in Japan, however it is approved only for research use in many countries other than Japan including Europe and North America. Also, it should be pronounced that serum SP-D might be beneficial for patients outside Japan if the test were approved for clinical use.*

**Response:** As the reviewer indicated, actually, the measurement of SP-D is not yet available at clinical practice in many countries. In the future, however, it should be validated whether the measurement of serum SP-D levels is beneficial for prediction of outcomes in patients with PM/DM-associated ILD if the test were approved for clinical use in many countries over the world.

To emphasize this issue, we added the following sentences as below.

In line 237 to 244 on page 10,

“SP-D can be measured as a laboratory test for clinical use in Japan based on the unique Japanese health insurance system. The measurement of SP-D is not yet available at clinical practice in many countries. Further study is required to investigate…, and to validate whether the measurement of serum SP-D levels is beneficial for prediction of outcomes in each MSA among patients with PM/DM-associated ILD in daily practice if the test is approved for clinical use in many countries over the world.”

*#3 Please enrich discussion about reasons for the difference in serum SP-D levels by MSA. Especially, focusing on mechanisms of translocation of SP-D molecules from alveolar epithelium to serum and on difference in damage of peripheral pulmonary structure by MSAs would be preferable.*

**Response:** Thank you for your constructive suggestion. Histopathological analysis was not conducted in our study. As the reviewer indicated, we speculate that the histopathological difference of ILD at diagnosis may contribute to the behavior of serum SP-D levels in each MSA. Furthermore, we analyzed disease duration and severity of pulmonary dysfunction with %vital capacity among the three groups, as the reviewer#1 suggested. The disease duration at diagnosis is significantly shorter in anti MDA-5 antibody-positive group than in anti-ARS antibody-positive group or double negative group. In terms of %vital capacity, the median value (interquartile range [IQR]) of %vital capacity was lower in anti-ARS antibody positive group: 70.2% (60-85), than in double-negative group: 84.9% (66-98) (*P* = 0.0002) or anti-MDA5 antibody positive group: 81% (66-98) (*P* = 0.0005) (revised Figure 2B). On the other hand, there was no significant difference (*P* = 0.53) between double-negative group and anti-MDA5 antibody-positive group. These findings might indicate that chronic damage and remodelling of peripheral pulmonary structure was more apparent in anti-ARS antibody-positive group, causing translocation of SP-D molecules from alveolar epithelium to serum much more.

To describe this point, we have added the following sentences as below.

In line 168 to 182 on page 7 to 8,

“We also compared disease duration and pulmonary function among the three groups. With regard to disease duration at diagnosis of PM/DM-associated ILD, the median disease duration (IQR) was significantly shorter in anti-MDA5 antibody-positive group: 2 months (1-3), than in anti-ARS antibody-positive group: 3 months (1-8) (*P* = 0.0002) or double-negative group: 3 months (1-6) (*P* = 0.0005). The range of disease duration in anti-ARS antibody-positive group was 0-122 months; longer than that of double-negative group with 0-88 months, although there was no significant difference of the median disease duration (*P* = 0.82) between the two groups. In terms of the severity of pulmonary dysfunction with %vital capacity (VC), the median value (IQR) of %VC was lower in anti-ARS antibody positive group: 70.2% (60-85), than in double-negative group: 84.9% (66-98) (*P* = 0.0002) or anti-MDA5 antibody positive group: 81% (66-98) (*P* = 0.0005) (Figure 2B). On the other hand, there was no significant difference (*P* = 0.53) between anti-MDA5 antibody-positive group and double-negative group. These results indicated more widespread damage of ILD as well as longer disease duration in anti-ARS antibody-positive group than the other two groups.”

In line 222 to 229 on page 9,

“Histopathological differences of ILD at diagnosis of PM/DM-associated ILD might contribute to the behavior of serum SP-D levels in each MSA group. In this study, the disease duration was significantly longer in in anti-ARS antibody-positive group or double-negative group than in anti-MDA5-positive group. In addition, the median value of %vital capacity was lower in anti-ARS antibody-positive group than in double-negative group or anti-MDA5 antibody-positive group. These findings might indicate that chronic damage and remodelling of peripheral pulmonary structure was more apparent in anti-ARS antibody-positive group, causing translocation of SP-D molecules from alveolar epithelium to blood vessels much more.”
